# Does Body Image Affect Quality of Life?: A Population Based Study

**DOI:** 10.1371/journal.pone.0163290

**Published:** 2016-09-20

**Authors:** Tufan Nayir, Ersin Uskun, Mustafa Volkan Yürekli, Hacer Devran, Ayşe Çelik, Ramazan Azim Okyay

**Affiliations:** 1 Administrative Department, Mersin Public Health Directorate, Mersin, Turkey; 2 Department of Public Health, Süleyman Demirel University Faculty of Medicine, Isparta, Turkey; 3 Department of Communicable Diseases, Ceyhan Community Health Center, Adana, Turkey; National Institute of Health, ITALY

## Abstract

Body image (BI) can be described as the assessment of both positive and negative emotion for one’s own body parts and their characteristics by himself or herself. Current research has concentrated mostly on the status of negative BI as a risk factor for mental health problems rather than as a public health problem, thereby little is known about the effects of BI on quality of life. Thus, the purpose of this study was to assess the BI and Quality of Life (QoL) of individuals and to investigate the relationship between the two. Individuals over 15 living in Isparta city center constitute the universe of this cross-sectional analytical study, carried out in 2014. The BI of individuals was measured by the Body Image Scale and The QoL of individuals was measured using the World Health Organization (WHO) Quality of Life Scale Short Form. The mean age of the participants was 31.9 ± 13.0 and 56.0% were female, 36.8% were married and 81.7% had education above high school. 25.7% had at least one chronic disease and 17.7% received medication regularly. Having good-very good health perception, having higher income than expenses, making regular exercises were predictors in enhancing the quality of life in certain aspects, however having a good body image came out as a predictor enhancing the quality of life in all sub-domains. BI was found closely related with QoL in all sub-domains. Our findings suggest that greater attention should be to be given to BI as a strong predictor of QoL.

## Introduction

Body image (BI) can be described as the assessment of both positive and negative emotion for one’s own body parts and their characteristics by himself or herself [1]. BI is a complicated construct that is composed of several components such as mental and emotional components, perceptual components and behavioral components [2].

It is well documented that a negative BI is associated with a range of adverse health outcomes, including low self-esteem, depressive mood and eating disorder symptoms [3,4].

However attention has concentrated mostly on the status of negative BI as a risk factor for mental health problems rather than as a public health problem in its own right, thereby little is known about the effects of BI on quality of life (QoL) [5].

The World Health Organization (WHO) defines QoL as the individual’s assessment of their position in life in the scope of culture and values, considering their goals, expectations, standards and concerns [6].

The concept of life quality closely related to health status, is one of the topics that is paid much attention by a large group of researchers engaged in the field of medicine and found worthy to investigate. The concept of life quality related to health, alternative explanations exist though, means “the perception of health and illness experience from the individuals’s point of view” [6,7].

As mentioned above not much attention was given to BI in terms of QoL. To the best of our knowledge, in Turkey, there is no previously conducted study investigating the association between BI and QoL. Therefore, the goal of this study is to assess the BI and QoL of individuals over 15 living in a city center and to investigate the relationship between the two.

## Materials and Methods

### Study Design

Individuals over 15 living in Isparta city center constitute the universe of this study which is a cross-sectional analytical study, carried out in 2014 (n:175.409). The sample size was calculated as 638 with an obesity prevalence of 30% with a 5% margin of error in OpenEpi Program (Open Source Epidemiologic Statistics for Public Health, Version 3.01, 2013). Using the cluster sampling method we reached a total of 650 people in 26 clusters and 25 people in each cluster.

The inclusion criterias for enrollment are to volunteer to participate in the study and to be over 15 years old. The exclusion criteria is to have mental retardation.

### Data collection instruments

Socio-demographic characteristics (age, gender, education level, economical perception, having a chronic disease, smoking, drinking alcohol, doing sport, weight, height) and other characteristics (the thoughts and requests about the weight of himself/herself, family, friends and spouse/partner, the status of using any method to lose weight at the time of survey and in the past year, the status of skipping meals and snacking between meals etc.) were collected with a questionnaire prepared by authors and included 28 items.

The BI of individuals was measured by the Body Image Scale developed by Secord and Jourard [8] and adapted to Turkish by Hovardaoglu [9]. Body Image Scale consists of 40 articles aiming to measure the level of satisfaction of various body functions and various parts of the body of individuals. This scale is a quinary Likert-type scale evaluated from the total score obtained from the scale and can get a score ranging from 40 to 200. Higher scores got by an individual indicates a higher level of satisfaction of individual’s own body. The QoL of individuals was measured using the Turkish version (TR) of WHO Quality of Life Scale Short Form (WHOQOL-BREF) [10]. WHOQOL-BREF (TR), a type of scale having the reliability and validity study [11,12], consists of 26 quinary Likert-type questions, two of which are general questions and the rest of which are questions about four different fields (physical, psychological, social and environment). Culture Standardized (CS) Environmental Area, which is obtained considering the answers to the 27th question added as a national question during the study for adaptation to Turkish, is an additional field used in national studies. The scale not having a total score, each area is evaluated independently and can have a value between 4–20 points. The increasing points for each area indicates the increasing QoL for this field. In this study, scores of the QoL were calculated for all fields of WHOQOL-BREF (TR) scale.

Data was gathered by making surveys prepared by researchers using face to face interview method.

### Statistical analysis

The dependent variables of the study were the scores of QoL and BI. Age, gender, education level, economical perception, having a chronic disease, smoking, drinking alcohol, doing sport, body mass index (BMI), the thoughts and requests about the weight of himself/herself, family, friends and spouse/partner, the status of using any method to lose weight at the time of survey and in the past year, the status of skipping meals and snacking between meals were independant variables. Data was evaluated using descriptive statistics, t-test, Pearson and Spearman correlation and linear regression analysis in computer. Statistical Package for the Social Sciences soft-ware (SPSS, Version 9.0, Inc. California, 1999) was used for all the statistical calculations.

### Ethical considerations

This study was conducted in accordance with the ethical standards of the Declaration of Helsinki, which promotes respect for all human beings and protects their health and rights. The Ethics Committee of Süleyman Demirel University Faculty of Medicine approved this study.

After informing the participants about the purpose of the trial (investigation, research, study), and where and how the obtained data would be used, written consents were obtained. The written consent was a separate standard document prepared according to the ethics committee suggestion. The participants of this study were over 15 years old. For over 18, written informed consent was provided by the participant ownself and for between 16–18 the written informed consent to participate was provided by his/her legal representative.

## Results

The mean age of the study group was 31.9 ± 13.0 and 56.0% were female, 36.8% were married and 81.7% had education of high school or above. 70.5% perceived their income as middle income (Table 1).

**Table 1:** Socio-demographic characteristics and body image of the study group and the distribution of WHOQOL-BREF (TR) sub-parameters according to these characteristics.

				Body Image Score (Mean±SD)	WHOQOL-BREF (TR) parameters (Mean±SD)				
Characteristics		Number	%		Physical domain	Psychological domain	Social domain	Environmental domain	CS environmental domain
Gender	Male	286	44	157,4 ± 23,2***	15,8 ± 2,7**	14,9 ± 2,8***	15,3 ± 3,2**	14,9 ± 2,5***	14,1±4,0
	Female	364	56	147,9 ± 23,8	15,1 ± 2,8	14,1 ± 2,7	14,5 ± 3,0	14,1 ± 2,5	13,6±4,3
Marital status	Single/widowed	411	63,2	153,1 ± 23,8	15,7 ± 2,7**	14,6 ± 2,8	15,0 ± 3,2	14,7 ± 2,6*	13,6±4,2
	Married	239	36,8	150,4 ± 24,3	15,0 ± 2,8	14,4 ± 2,5	14,6 ± 2,9	14,1 ± 2,3	14,2±4,1
Education	Primary school or less	119	18,3	146,1±24,5**	14,8±3,3*	13,8±2,7**	14,5±3,1	13,9±2,6**	13,4±4,3
	High school or above	531	81,7	153,5±23,7	15,6±2,6	14,7±2,7	14,9±3,1	14,6±2.4	13,9±4,2
Occupational status	Working	338	52	156,1 ± 24,0***	15,5 ± 2,8	14,8 ± 2,7**	14,9 ± 3,3	14,6 ± 2,5	14,3 ±4,1**
	Not working	312	48	147,8 ± 23,3	15,4 ± 2,7	14,2 ± 2,7	14,8 ± 2,9	14,4 ± 2,5	13,4±4,3
Economic perception	Good or very good	85	13,0	160,9±24,1***	16,4±2,4***	15,7±3,0***	15,6±3,1*	16,2±2,3***	15,0±3,8**
	Moderate or worse	565	87	150,8±23,7	15,3±2,8	14,3±2,7	14,7±3,1	14,2±2,4	13,7±4,2
Income balance	Income > Expenses	170	26,2	157,2±23,6***	16,1±2,6***	15,4±2,7***	15,2±2,9***	15,7±2,3***	14,8±4,1***
	Income = Expenses	389	59,8	152,0 ± 23,2	15,4 ± 2,6	14,4 ± 2,5	15,0 ± 3,1	14,3 ± 2,3	13,9±4,1
	Income < Expenses	91	14	143,3 ± 25,4	14,4 ± 3,3	13,1 ± 3,1	13,5 ± 3,1	13,1 ± 2,6	11,9±4,1
**Total**		650	100	152,1 ± 24,0	15,4 ± 2,8	14,5 ± 2,7	14,8 ± 3,1	14,5 ± 2,5	13,8±4,2

Mean±SD: Mean±Standart Deviation, CS: Culture Standardized, WHOQOL-BREF(TR): Turkish Version (TR) of World Health Organization Quality of Life Scale Short Form.

* p<0,05

** p<0,01

*** p<0,001

31.1% were smoking, 28.8% were drinking alcohol and 22.0% were making regular exercise. The average BMI of the study group was 24.0±4.4 and 36.0% were fat or overwight (BMI ≥25kg/m2). 25.7% had at least one chronic disease and 17.7% received medication regularly. 71.5% were skipping at least one meal during the day (Table 2). The most skipped meal was lunch (49.0%). 84.2% of the group were snacking between meals and the most preferred snack was fruit with 49.7%.

**Table 2:** Characteristics of health status and body image of the study group and the distribution of WHOQOL-BREF (TR) sub-parameters according to these characteristics.

				Body Image Score (Mean±SD)	WHOQOL-BREF (TR) parameters (Mean±SD)				
Characteristics		Number	%		Physical domain	Psychological domain	Social domain	Environmental domain	CS environmental domain
Health perception	Good or very good	450	69,2	156,9±21,9***	16,1±2,4***	15,0±2,6***	15,2±2,9***	14,9±2,4***	14,1±4,1**
	Moderate or worse	200	30,8	141,4±25,1	13,8±3,0	13,3±2,7	13,9±3,3	13,5±2,4	13,2±4,2
BMI (kg/m^2)^	Under 18	24	3,7	148,1±22,9	15,0±3,4*	14,5±2,9	14,6±2,7	14,3±3,1	13,3±4,0
	18–24,9	392	60,3	153,0±23,4	15,7±2,5	14,6±2,7	15,0±3,1	14,6±2,4	13,7±4,3
	25 and above	234	36	151,0±25,0	15,0±3,1	14,4±2,7	14,6±3,1	14,4±2,5	14,1±4,0
Smoking	No	448	68,9	149,8±23,2***	15,4±2,7	14,3±2,7*	14,7±3,0*	14,4±2,4	13,8±4,1
	Yes	202	31,1	157,2±24,9	15,5±2,8	14,9±2,8	15,2±3,4	14,7±2,6	14,0±4,4
Alcohol consumption	No	463	71,2	150,6±24,1*	15,2±2,9**	14,3±2,7**	14,5±2,9***	14,3±2,4**	13,8±4,2
	Yes	187	28,8	155,9±23,2	15,9±2,5	15,0±2,7	15,7±3,4	14,9±2,6	13,8±4,2
Making regular exercise	Yes	143	22	161,1±21,3***	16,5±2,4***	15,4±2,8***	15,7±2,9***	15,3±2,4***	14,3±4,6
	No	507	78	149,6±24,1	15,1±2,8	14,2±2,7	14,6±3,1	14,3±2,5	13,7±4,1
Having a chronic disease	No	483	74,3	154,4±23,8***	15,9±2,5***	14,7±2,6***	15,0±3,1	14,6±2,5	14,1±4,1**
	Yes	167	25,7	145,6±23,5	14,2±3,2	13,8±2,9	14,5±3,2	14,2±2,4	13,0±4,3
Receiving medication regularly	No	535	82,3	154,3±23,7***	15,8±2,5***	14,7±2,6***	15,0±3,1**	14,6±2,5*	14,0±4,2*
	Yes	115	17,7	142,2±23,1	13,8±3,3	13,4±2,9	14,0±3,2	14,0±2,4	13,1±3,9
Skipping meals	No	185	28,5	155,1±25,1*	15,7±3,1	15,0±2,7**	15,0±3,3	14,9±2,5**	14,4±3,9*
	Yes	465	71,5	150,9±23,5	15,3±2,6	14,3±2,7	14,8±3,0	14,3±2,4	13,6±4,3
Diet history in the past year	No	438	67,4	153,7±23,7*	15,5±2,8	14,6±2,8	14,9±3,0	14,5±2,5	14,2±4,1**
	Yes	212	32,6	148,7±24,2	15,2±2,7	14,2±2,7	14,7±3,3	14,5±2,4	13,1±4,3
**Total**		650	100	152,1±24,0	15,4±2,8	14,5±2,7	14,8±3,1	14,5±2,5	13,8±4,2

Mean±SD: Mean±Standart Deviation, CS: Culture Standardized, BMI: Body Mass Index, WHOQOL-BREF(TR): Turkish Version (TR) of World Health Organization Quality of Life Scale Short Form.

* p<0,05

** p<0,01

*** p<0,001

### Specific Results on BI

The average of BI Score of the research group was 152.1±24.0. (Table 3). The average of BI Scores of women, those with a chronic disease, those using medication, those skipping meals, those having the opinion that they are not normal by their family, friends, spouse and himself/herself, those having the consideration that they need to make changes in their weight by their family, friends, spouse and himself/herself and those having the history of dieting in the past year were lower than others.(p<0.001, p<0.001, p<0.001, p<0.05, p<0.001, p<0.001, p<0.001, p<0.001, p<0.001, p<0.001, p<0.001 and p<0.001 respectively) (Tables 2 and 4).

**Table 3:** Correlation of age, number of applications to health institutions and body image scores of the study group with WHOQOL-BREF (TR) sub-parameters.

		WHOQOL-BREF (TR) parameters (Mean±SD)				
Characteristics	Mean±SD	Physical domain	Psychological domain	Social domain	Environmental domain	CS environmental domain
Age (years)	31,9±13	-0,220***	-0,091*	-0,101*	-0,100*	0,028
Number of applications to health organizations	3,7±5,4	-0,251***	-0,178***	-0,056	-0,110**	-0,050
Body Image Score	152,1±24,0	0,478***	0,584***	0,495***	0,482***	0,245***

Mean±SD: Mean±Standart Deviation, CS: Culture Standardized, r: Pearson correlation coefficient, WHOQOL-BREF(TR): Turkish Version (TR) of World Health Organization Quality of Life Scale Short Form.

* p<0,05

** p<0,01

*** p<0,001

**Table 4:** Social pressure factors and body image of the study group and the distribution of WHOQOL-BREF (TR) sub-parameters according to these characteristics.

				Body Image Score (Mean±SD)	WHOQOL-BREF (TR) parameters (Mean±SD)				
Social pressure factors related to one’s physical appearance		Number	%		Physical domain	Psychological domain	Social domain	Environmental domain	CS environmental domain
Family’s thought	Normal	354	54,5	155,9±23,1***	15,7±2,6**	14,8±2,7**	15,0±3,1	14,7±2,4*	14,2±4,1**
	Not normal	296	45,5	147,6±24,3	15,1±2,9	14,2±2,7	14,7±3,2	14,3±2,5	13,3±4,2
Family’s desire	Should remain the same	371	57,1	156,0±23,3	15,7±2,7	14,8±2,7	15,0±3,2	14,7±2,5	14,4±4,1
	Should change	279	42,9	146,9±23,9***	15,1±2,8**	14,1±2,7**	14,6±3,0	14,2±2,4**	13,2±4,2***
Friends’ thought	Normal	404	62,2	156,6±23,1***	15,9±2,6***	14,9±2,7***	15,2±3,2***	14,9±2,5***	14,3±4,2**
	Not normal	246	37,8	144,8±23,7	14,7±3,0	13,8±2,7	14,3±2,9	14,0±2,4	13,1±4,0
Friends’ desire	Should remain the same	431	66,3	156,2±22,7***	15,9±2,6***	14,9±2,7***	15,1±3,2**	14,8±2,5***	14,3±4,1***
	Should change	219	33,7	144,0±24,5	14,6±2,9	13,7±2,6	14,3±3,0	13,9±2,4	12,9±4,3
Spouse’s thought	Normal	435	66,9	155,4±22,9***	15,8±2,6***	14,9±2,6***	15,1±3,2**	14,8±2,4***	14,1±4,2*
	Not normal	215	33,1	145,5±24,7	14,7±3,0	13,7v2,8	14,3±2,9	14,0±2,5	13,3±4,1
Spouse’s desire	Should remain the same	436	67,1	155,1±23,2***	15,8±2,7***	14,8±2,7***	15,0±3,2	14,7±2,5**	14,1±4,1**
	Should change	214	32,9	146,0±24,4	14,7±2,9	13,8±2,7	14,5±2,9	14,1±2,4	13,2±4,3
Own thought	Normal	390	60	158,6±21,5***	15,9±2,6***	15,0±2,7***	15,1±3,1**	14,8±2,5**	14,3±4,2**
	Not normal	260	40	142,4±24,3	14,7±2,9	13,7±2,7	14,4±3,0	14,1±2,3	13,2±4,1
Own desire	Should remain the same	349	53,7	159,4±21,9***	15,9±2,7***	15,0±2,7***	15,2±3,2**	14,8±2,6**	14,3±4,1**
	Should change	301	46,3	143,6±23,5	14,9±2,8	13,9±2,6	14,4±3,0	14,2±2,3	13,3±4,3
**Total**		650	100	152,1±24,0	15,4±2,8	14,5±2,7	14,8±3,1	14,5±2,5	13,8±4,2

Mean±SD: Mean±Standart Deviation, CS: Culture Standardized, WHOQOL-BREF(TR): Turkish Version (TR) of World Health Organization Quality of Life Scale Short Form.

* p<0,05

** p<0,01

*** p<0,001

Being a woman decreased the BI score of 3.7 units, the individual’s own desire to make changes in body weight decreased the BI score of 7.7 units (p<0.05 and p<0.01 respectively). Working increased the BI score of 4.5 units, having good-very good economical sense increased the BI score of 6.8 units, having good-very good health perception increased the BI score of 9.3 units, smoking increased the BI score of 5.7 units and making regular exercise increased the BI score of 6.5 units. (p<0.05, p<0.05, p<0.001, p<0.01 and p<0.01 respectively) (Table 5).

**Table 5:** Regression analysis of the variables that are found to be associated in the univariate analysis with sub-parameters of WHOQOL-BREF (TR).

Variables included in the analysis	Body Image	WHOQOL-BREF (TR) parameters (Exp [B] (%95 Confidence Interval))				
		Physical domain	Psychological domain	Social domain	Environmental domain	CS environmental domain
Age (years)	-0,06 (-0,21–0,10)	-0,02 (-0,04–0,03)	0,01 (-0,01–0,02)	-0,01 (-0,02−0,01)	0,00 (-0,02–0,02)	a
Gender (female = 1, male = 0)	-3,68 (-7,34– -0,02)*	-0,13 (-0,52–0,26)	-0,02 (-0,40–0,35)	-0,10 (-0,56−0,36)	-0,15 (-0,51–0,20)	a
Marital status (married = 1, other = 0)	a	-0,10 (-0,57–0,38)	a	a	-0,44 (-0,87– -0,01)*	a
Education (high school or above = 1, other = 0)	0,90 (-3,81–5,61)	-0,33 (-0,83–0,16)	0,26 (-0,23–0,74)	a	0,15 (-0,31–0,60)	a
Occupational status (working = 1, other = 0)	4,49 (0,94–8,03)*	A	-0,16 (-0,53–0,21)	a	a	0,39 (-0,25–1,04)
Economic perception (very good-good = 1, other = 0)	6,80 (1,34–12,26)*	0,31 (-0,28–0,91)	0,29 (-0,28–0,85)	0,14 (-0,49–0,76)	0,83 (0,28–1,37)**	0,48 (-0,57–1,54)
Income balance (income>expenses = 1, other = 0)	0,25 (-4,01–4,52)	0,20 (-0,26–0,66)	0,56 (0,12–1,00)*	a	0,81 (0,39–1,22)***	0,62 (-0,19–1,43)
Health perception (very good-good = 1, other = 0)	9,27 (5,29–13,25)***	1,11 (0,68–1,55)***	0,56 (0,14–0,98)**	0,24 (-0,26–0,74)	0,53 (0,14–0,93)**	<-0,01 (-0,75–0,75)
BMI (≥25 kg/m^2^ = 1, other = 0)	a	0,04 (-0,41–0,49)	a	a	a	a
Number of applications to health organizations	-0,30 (-0,63–0,03)	-0,05 (-0,08–0,01)*	-0,03 (-0,06–0,01)	a	<-0,01 (-0,04–0,03)	a
Smoking (yes = 1, other = 0)	5,74 (1,65–9,84)**	A	0,15 (-0,28–0,57)	-0,32 (-0,83–0,20)	a	a
Alcohol consumption (yes = 1, other = 0)	-2,62 (-6,78–1,54)	0,25 (-0,17–0,66)	0,20 (-0,23–0,63)	1,02 (0,49–1,55)***	0,29 (-0,08–0,66)	a
Making regular exercise (yes = 1, other = 0)	6,54 (2,50–10,59)**	0,65 (0,21–1,08)**	0,27 (-0,15–0,69)	0,43 (-0,08–0,95)	0,29 (-0,11–0,69)	a
Having a chronic disease (yes = 1, no = 0)	1,58 (-4,28–7,44)	-0,36 (-0,99–0,27)	0,08 (-0,53–0,69)	a	a	-0,92 (-2,03–0,19)
Receiving medication regularly (yes = 1, no = 0)	-4,47 (-11,30–2,36)	-0,33 (-1,07–0,41)	-0,42 (-1,13–0,29)	-0,10 (-0,72–0,52)	0,19 (-0,30–0,68)	0,35 (-0,94–1,64)
Skipping meals (yes = 1, no = 0)	-0,89 (-4,69–2,91)	A	-0,40 (-0,80– -0,01)*	a	-0,36 (-0,73–0,02)	-0,48 (-1,19–0,23)
Diet history in the past year (yes = 1, no = 0)	2,40 (-1,57–6,35)	A	a	-0,09 (-0,59–0,42)	a	a
Family’s thought (not normal = 1, normal = 0)	3,12 (-2,18–8,42)	0,43 (-0,14–1,01)	0,42 (-0,13–0,97)	a	0,35 (-0,18–0,87)	0,02 (-1,00–1,03)
Family’s desire (Should change = 1, Should remain the same = 0)	-1,74 (-6,88–3,40)	0,22 (-0,34–0,77)	0,12 (-0,41–0,65)	a	-0,12 (-0,63–0,39)	-0,60 (-1,59–0,39)
Friends’ thought (not normal = 1, normal = 0)	-3,99 (-9,57–1,60)	-0,09 (-0,70–0,51)	-0,14 (-0,72–0,44)	-0,33 (-0,99–0,34)	0,02 (-0,53–0,57)	0,04 (-1,03–1,11)
Friends’ desire (Should change = 1, Should remain the same = 0)	-1,28 (-7,09–4,53)	-0,53 (-1,16–0,10)	-0,20 (-0,80–0,40)	0,17 (-0,51–0,85)	-0,48 (-1,06–0,09)	-0,85 (-1,97–0,26)
Spouse’s thought (not normal = 1, normal = 0)	-3,47 (-9,54–2,60)	-0,40 (-1,05–0,26)	-0,34 (-0,97–0,29)	-0,14 (-0,67–0,40)	-0,35 (-0,95–0,25)	0,36 (-0,81–1,53)
Spouse’s desire (Should change = 1, Should remain the same = 0)	2,62 (-3,62–8,85)	-0,26 (-0,94–0,42)	-0,27 (-0,92–0,38)	a	0,10 (-0,52–0,71)	-0,19 (-1,39–1,01)
Own thought (not normal = 1, normal = 0)	-5,05 (-10,63–0,54)	-0,23 (-0,83–0,38)	-0,26 (-0,84–0,32)	0,60 (-0,11–1,30)	0,06 (-0,49–0,62)	-0,19 (-1,26–0,88)
Own desire (Should change = 1, Should remain the same = 0)	-7,71 (-13,46–-1,97)**	0,45 (-0,16–1,05)	0,39 (-0,19–0,97)	0,13 (-0,58–0,85)	0,54 (-0,01–1,09)	0,48 (-0,57–1,54)
Body image	a	0,04 (0,03–0,05)***	0,06 (0,05–0,07)***	0,06 (0,05–0,07)***	0,04 (0,04–0,05)***	0,03 (0,02–0,05)***
Constatnt	151,57 (142,22–160,92)***	9,76 (8,15–11,38)***	5,22 (3,66–6,79)***	5,00 (3,25–6,75)***	7,38 (5,89–8,87)***	9,17 (6,73–11,6)***
Adjusted R^2^	0,24***	0,34***	0,37***	0,26***	0,31***	0,07***

a: Variable not included in the model, CS: Culture standardized, WHOQOL-BREF(TR): Turkish Version (TR) of World Health Organization Quality of Life Scale Short Form.

* p<0,05

** p<0,01

*** p<0,001

### Specific Results on QoL

The mean scores of WHOQOL-BREF (TR) regarding physical, psychological, social, environment and CS environmental areas were 15.4±2.8, 14.5±2.7, 14.8±3, 14.5±2.5 and 13.8±4.2 respectively. (Table 3).

Physical domain scores were significantly lower for women, the married, the fat/overweight according to BMI, patients with chronic diseases, those using drugs, those having the opinion that they are not normal by their family, friends, spouse and himself/herself, those having the consideration that they need to make changes in their weight by their family, friends, spouse and himself/herself (p<0.01, p<0.01, p<0.01, p<0.001, p<0.001, p<0.001, p<0.01, p<0.01, p<0.001, p<0.001, p<0.001, p<0.001, p<0.001 and p<0.001 respectively) (Tables 2 and 4). Physical domain scores were higher for those having higher education than high school, those having good-very good economical sense, those having good-very good health perception, those drinking alcohol and those making regular exercises (p<0.05, p<0.001, p<0.001, p<0.001, p<0.01 and p<0.001 respectively) (Tables 1 and 2).

Psychological domain scores were significantly lower for women, patients with chronic diseases, those using drugs, those having the opinion that they are not normal by their family, friends, spouse and himself/herself, those having the consideration that they need to make changes in their weight by their family, friends, spouse and himself/herself (p<0.001, p<0.001, p<0.001, p<0.01, p<0.01, p<0.001, p<0.001, p<0.001, p<0.001, p<0.001 and p<0.001 respectively) (Tables 2 and 4). Psychological domain scores were higher for those having higher education than high school, those working, those having good-very good economical sense, those having good-very good health perception, those drinking alcohol and those making regular exercises (p<0.01, p<0.01, p<0.001, p<0.001, p<0.05, p<0.01 and p<0.001) (Tables 1 and 2).

Social domain scores were significantly lower for women, those having the opinion that they are not normal by their family, friends, wife and himself/herself, those having the consideration that they need to make changes in their weight by their family, friends, spouse and himself/herself (p<0.01, p<0.001, p<0.01, p<0.001 and p<0.01 respectively) (Tables 2 and 4). Social domain scores were higher for those having good-very good economical sense, those drinking alcohol, smoking and those making regular exercises (p<0.01, p<0.001, p<0.05, p<0.001 and p<0.001 respectively) (Tables 1 and 2).

Environmental domain scores were significantly lower for women, the married, those using drugs, those skipping meals, those having the opinion that they are not normal by their family, friends, spouse and himself/herself, those having the consideration that they need to make changes in their weight by their family, friends, spouse and himself/herself (p<0.001, p<0.05, p<0.01, p<0.05, p<0.01, p<0.001, p<0.001, p<0.001, p<0.01, p<0.01 and p<0.01 respectively) (Tables 2 and 4). Environmental domain scores were higher for those having higher education than high school, those having good-very good economical sense, those having good-very good health perception, those having more income than expenses, those drinking alcohol and those making regular exercises (p<0.01, p<0.001, p<0.001, p<0.001, p<0.01 and p<0.001 respectively) (Tables 1 and 2).

CS environmental domain scores were significantly lower for patients with chronic diseases and those using drugs, those skipping meals, those having the opinion that they are not normal by their family, friends, spouse and himself/herself, those having the consideration that they need to make changes in their weight by their family, friends, spouse and himself/herself (p<0.01, p<0.05, p<0.05, p<0.01, p<0.01, p<0.05, p<0.01, p<0.001, p<0.001, p<0.01, p<0.01 and p<0.01 respectively) (Tables 2 and 4).

As age and number of applications for health organizations increased, physical, psychological and environmental domain scores reduced (for age; p<0.001, p<0.05 and p<0.05 respectively, for applications for health organizations; p<0.001, p<0.001 and p<0.01 respectively). Furthermore, as age increased, social domain scores decreased (p<0.05). As BI score increased, QoL scores in five sub-areas also increased (for all p<0.001) (Table 3).

### Regression analysis results

As a result of regression analysis of variables associated with the univariate analysis with sub parameters of WHOQOL-BREF (TR) (Table 5);

Having good-very good health perception increased physical domain score by 1.1 units, making regular exercises increased physical domain score by 0.7 units (p<0.001 and p<0.01 respectively). A unit increase in applications for health organizations reduced physical domain score by 0.1 units (p<0.05).

Having higher income than expenses increased psychological domain score by 0.6 units, having good-very good health perception increased psychological domain score by 0.6 units (p<0.05 and p<0.01 respectively). Skipping meals during the day reduced psychological domain score by 0.4 units (p<0.05).

Drinking alcohol increased social domain score by 1.0 units (p<0.001).

Being married reduced environmental domain score by 0.4 units (p<0.05). Having good-very good economical perception increased environmental domain score by 0.8 units, having higher income than expenses increased environmental domain score by 0.8 units and having good-very good health perception increased environmental domain score by 0.5 units (p<0.01, p<0.001 and p<0.01 respectively).

A unit increase in BI score results in 0.1 unit increased for every five domains (for all p<0.001).

## Discussion

This study revaled that BI significantly affects the QoL in every sub-domain. It is very important to create a positive BI perception to improve the QoL of individuals. When risk groups and risk factors associated with negative BI is known, particular attention may be given to these groups. Similarly, knowing the factors which positively affect BI may give hints for possible interventions. The findings of this study provide us important evidence on this aspect. Being a woman affects BI negatively, making women a risk group. In this respect steps should be taken to ensure a positive BI in females. Individuals who have a desire to change in terms of BI, are also a risk group. BI is positive in the individuals with a good or very good health perception. Also, making regular exercises was found to improve BI. Thus, regular exercise programs that positively affect health perception should be encouraged.

In this study we assessed the factors that are independently associated with BI perception and QoL among individuals over 15 living in Isparta city center, while controlling for all other factors. Below, only the factors that displayed significant associations in the regression analysis were discussed.

Anticipated relation between gender and BI perception is verified by the results of this study: females tended to have a more negative BI perception compared to males. This is supported by other studies showing that women were more likely to perceive themselves as being overweight than men [13,14]. At this point it should be noted that in women, many unhealthy attitudes such as bulimia and anorexia are the results of dissatisfaction with self image, especially dissatisfaction with aspects associated to body weight [15].

We found that working and making regular exercises increased the BI score. Positive effects of exercise on BI have been firmly established in the literature [16–19]. There is also evidence that exercise improves BI, even though body weight and shape do not change [20]. However, exercising compulsively and excessively is a prevalent purging strategy used to make up for caloric intake or to alter one’s body weight, size, or shape, resulting in eating disorders related to body dissatisfaction, thus this fact should be considered while appraising exercise in regards to BI [21].

Having good-very good economical sense was found to increase the BI score in this study. Likewise, in a study conducted in Brazil in 2011 people with lower economic status were reported to be more dissatisfied with their current body silhouette [22]. However this result is controversial in the literature since some say BI dissatisfaction was most evident among people of higher socioeconomic classes [23]. The diversities in these studies are thought to be due to the differences in the methodologies.

In this study, it is found that having good-very good health perception increased the BI score. There are studies in line with our study, reporting body dissatisfaction was associated with the increased likelihood of impairment for certain aspects of health [24,25]. It is thought that personality characteristics related to body dissatisfaction, such as low self-esteem, depressive mood and perfectionism, may promote negative evaluation of physical health [26].

Interestingly we found that smoking increased the BI score. Although some local studies are in accordance with our results [27,28], it is widely accepted that smoking is associated with poor BI [29–32]. We believe that smoking may improve BI as a coping skill, however further research on this subject should be carried out.

In our study having good-very good health perception was a predictor in enhancing the QoL in physical, psychological and environmental domains. In the literature, it is well documented that several health problems, especially the chronic conditions are associated with a decreased QoL [33–35]. It is stated that subjective health parameters could be more significant factors of life satisfaction than objective ones [36]. Thus, it is not surprising that having a better health sense improves QoL.

We found that having higher income than expenses was a predictor in enhancing the QoL in psychological and environmental domains and having good-very good economical sense was a predictor in enhancing the QoL in environmental domain. Although there are studies in line with our study [37,38], the issue is inconclusive in the literature. For example, research by Kenny [39] and Stewart [40] reported that in middle-income countries and across several European countries, there was little proof of relationship between economic development and gross domestic product per capita and subjective well-being. The relation described in the present research is considered to be the result of richer individuals’ having more access to social activities/services enhancing their social attendance and hence their QoL.

Our results demonstrated making regular exercises was a predictor in enhancing QoL only in the physical domain. Making exercises and physical activity have been shown to maintain good QoL in several studies [41,42]. There is a positive association between physical activity and perception of QoL, which varies according to the domains of QoL assessed [43]. Further studies should be encouraged to investigate the association between physical activity and exercise and the different domains of QoL.

We found that skipping meals during the day was reducing the QoL in psychological domain. Although there are numerous studies investigating the association between nutrition and QoL [44–46], we did not come across much evidence particularly regarding skipping meals. In a study from Mexico, however, low QoL was reported to be associated with skipping meals, which is in line with our results [47]. Since there is not sufficient evidence to discuss the association found in this study, we suggest further studies to be conducted.

In our study drinking alcohol was a predictor in enhancing QoL in the social domain. Some researches indicated a linear or inverse J-shaped relationship between QoL and alcohol use, in such a manner that at the greater levels of use of alcohol, which includes individuals diagnosed with alcohol use disorders, QoL is lower as compared to standard or low risk users and abstainers [48,49]. The association identified in the present study is thought to be due to the fact that alcohol consumption at a moderate level of may be positive in terms of stress relief and psychological health [50].

Another interesting result found in this study is that being married was reducing the QoL in environmental domain. In the literature considerable evidence points to the enjoyment of better health and QoL among married older adults relative to their non-married peers [51–53]. However, being in line with our study, there are studies reporting younger married people did not have better QoL than their non-married peers [54,55]. We believe that the negative impact of marriage on QoL is likely to be due to the younger sample in this study.

As a final result, this study demonstrated that having a good BI came out as a predictor enhancing the QoL in all sub-domains. In accordance with our study, Mond et al. reported higher levels of body dissatisfaction were associated with poorer QoL [5]. This finding is notable because interest in BI has principally focused on for more adverse outcomes, such as low self-esteem, depressive mood and eating disorders [56,57].

## Conclusions

In conclusion, as distinct from all of the other parameters used to assess QoL, BI was found closely related with QoL in all sub-domains. Our findings suggest that greater attention should be to be given to BI as a strong predictor of QoL. We expect that the data collected in this study will serve as a base for other researchers to investigate BI from a different point of view.

## Supporting Information

S1 File Dataset. (SAV)

S2 File Questionnaire. (DOC)
