# Supplementary material for: Does Body Image Affect Quality of Life?: A Population Based Study
Source: PLoS One. 2016 Sep 20;11(9):e0163290. doi: 10.1371/journal.pone.0163290 (PMC5029906; doi:10.1371/journal.pone.0163290)
Supplement: S2 File — (DOC) [file pone.0163290.s002.doc]

**SURVEY FORM**

Dear participants,

This survey form was designed to teach research planning to the last year students of Süleyman Demirel University Faculty of Medicine, Public Health Department and is a part of a study aiming to investigate the effect of body image on quality of life. The information obtained will be used for research purposes only and will not be shared for other purposes. Profile of the participants will be kept confidential. Thank you for your contributions.

Süleyman Demirel University Faculty of Medicine Department of Public Health

(0246 2113633)

**Neighborhood:** ………………………….  **Name of the applicator:**……………

**1- How old are you?** ..................

**2- What is your gender?** 1- Male 2- Female

**3- What is your marital status?** 1-Single 2-Married 3-Widowed

**4- What is your educational status?** 1-Illiterate 2-Literate 3-Primary school 4-Middle School

5-High School 6-Junior college 7- University

**5- Your job?** 1- Not working 2- Officer 3- Worker 4- Artisan 5- Self employed 6- Farmer 7- Other...........

**6- At which economic level you perceive yourself?**

1- Very high 2- High 3- Middle 4- Low 5- Very low

**7- Please select the most appropriate of the following options about your economic situation.**

(1) My revenue is more than my expenses, I can accumulate.

(2) My revenue is barely enough for my expenses, I can not accumulate.

(3) My revenue is less than my expenses, I have difficulties in making a living.

**8- How do you perceive your health?**

1- Very good 2- Good 3- Moderate 4- Bad 5- Very bad

**9- How tall are you?: ……….cm 10- What is your weight?: ……kg**

**11**- **How many times did you have applied to a health care provider in the last year?** ………….

**12- Do you have a chronic disease diagnosed by a physician?**

1- Yes (Name of the disease…………………..) 2- No

**13-** **Is there any medication that you are using continuously? (Please specify the name if available.)**

1- Yes – (Please write the name of medicine) ...................................................... 2- No

**14- Please select the most appropriate of the following options about your smoking status.**

1. **Non-smoker:** Never smoked.
2. **Smoker:** I smoke, including at least one every day.
3. **Quitted**: I smoke for a while before I left and I do not smoke.

**15- Please select the most appropriate of the following options about your alcohol consumption.**

1. I do not use alcohol.
2. Occasionally getting alcohol.
3. I frequently get alcohol..

**16- Please select the most appropriate of the following options about your exercise status.**

1. I do not do regular exercises
2. I rarely do exercises
3. I make at least 3 days a week for at least 20 minutes of exercise.

**17- Do you skip meals during the day?** 1- Yes 2- No

**18- Which meal do you usually skip?** 1- Breakfast 2- Lunch 3- Dinner

**19- Do you have snacking habit?** 1- Yes 2- No

**20- Which of the following do you prefer to snack between meals?**

1. Milk/Yogurt/Buttermilk/Dairy
2. Fruit/Vegetable
3. Wheel/Burgers/Other pastry
4. Biscuit/Wafer/Cakes/Pies/Desserts etc.
5. Chips/Popcorn etc.
6. Coke/Sodas
7. Chocolate
8. Other (Please specify……………………………….)

**21.** **What does your family think about your weight?**

( ) Very thin ( ) Thin ( ) Normal ( ) Overweight ( ) Very Overweight

**22. What is the desire of your family about your appearance?**

1- Does not want any change

2- Wants me to be thinner/slimmer

3- Wants me to put on weight

**23. What do your friends think about your weight?**

( ) Very thin ( ) Thin ( ) Normal ( ) Overweight ( ) Very Overweight

**24. What is the desire of your friends about your appearance?**

1- Does not want any change

2- Wants me to be thinner/slimmer

3- Wants me to put on weight

**25. What does your spouse/partner think about your weight?**

( ) Very thin ( ) Thin ( ) Normal ( ) Overweight ( ) Very Overweight

**26. What is the desire of your spouse/partner about your appearance?**

1- Does not want any change

2- Wants me to be thinner/slimmer

3- Wants me to put on weight

**27. What do you think about your weight?**

( ) Very thin ( ) Thin ( ) Normal ( ) Overweight ( ) Very Overweight

**28. How would you like your appearance to be?**

1- Do not want any change

2- Want to be thinner/slimmer

3- Want to put on weight

**29.Do you currently use a method to lose weight?** 1- Yes 2- No

**30.Did you make any effort to lose weight in the past year?** 1- Yes 2- No

**31.Which of the following methods did you apply to lose weight in the past year? (You can select more than one option)**

|  | Yes | No |
| --- | --- | --- |
| 1. I did regular exercises to lose weight. |  |  |
| 1. I did a diet to lose weight under dietician/physician control. |  |  |
| 1. I have used medicines under a physician control. |  |  |
| 1. I applied myself diet. |  |  |
| 1. I took diuretics to lose weight. |  |  |
| 1. I have used medicines that activate the intestines to lose weight. |  |  |
| 1. I have used herbal products (tea, etc.) for weight loss. |  |  |
| 1. I vomited after meals to lose weight. |  |  |
| 1. I skipped meals. |  |  |
| 10. Other (please specify…………………………………………….….) |  |  |

**BODY IMAGE SCALE**

**DESCRIPTION:** In this survey, what you need to do is assess your feelings about your body features according to the statement. It has been developed to determine feelings of people about their own bodies. There are several physical characteristics and like/dislike expressions below.

|  |  | | (5) I like very much | (4) I quite like | (3) Neutral | (2) I do not like much | (1) I do not like it at all |
| --- | --- | --- | --- | --- | --- | --- | --- |
| 1 | My hair | |  |  |  |  |  |
| 2 | My facial complexion | |  |  |  |  |  |
| 3 | My appetite | |  |  |  |  |  |
| 4 | My hands | |  |  |  |  |  |
| 5 | Distribution of hair over my body |  | |  |  |  |  |
| 6 | My nose | |  |  |  |  |  |
| 7 | My physical strength | |  |  |  |  |  |
| 8 | My urine feces order | |  |  |  |  |  |
| 9 | My muscle strength | |  |  |  |  |  |
| 10 | My waist | |  |  |  |  |  |
| 11 | My energy level | |  |  |  |  |  |
| 12 | My back | |  |  |  |  |  |
| 13 | My ears | |  |  |  |  |  |
| 14 | My age | |  |  |  |  |  |
| 15 | My chin | |  |  |  |  |  |
| 16 | My body build | |  |  |  |  |  |
| 17 | My profile | |  |  |  |  |  |
| 18 | My height | |  |  |  |  |  |
| 19 | Intensity of my feelings | |  |  |  |  |  |
| 20 | My endurance of pain | |  |  |  |  |  |
| 21 | Width of my shoulders | |  |  |  |  |  |
| 22 | My arms | |  |  |  |  |  |
| 23 | My chest | |  |  |  |  |  |
| 24 | My eyes | |  |  |  |  |  |
| 25 | My digestion system | |  |  |  |  |  |
| 26 | My hips | |  |  |  |  |  |
| 27 | My disease resistance | |  |  |  |  |  |
| 28 | My legs | |  |  |  |  |  |
| 29 | My teeth | |  |  |  |  |  |
| 30 | My sexual performance | |  |  |  |  |  |
| 31 | My feet | |  |  |  |  |  |
| 32 | My sleep order | |  |  |  |  |  |
| 33 | My voice | |  |  |  |  |  |
| 34 | My health | |  |  |  |  |  |
| 35 | My sex activities | |  |  |  |  |  |
| 36 | My knees | |  |  |  |  |  |
| 37 | My posture | |  |  |  |  |  |
| 38 | My face | |  |  |  |  |  |
| 39 | My weight | |  |  |  |  |  |
| 40 | My sexual organ | |  |  |  |  |  |

**WHOOQL – BREF (World Health Organization Quality Of Life Questionnaire Abbreviated Version)**

**Instructions:** This assessment asks how you feel about your quality of life, health, or other areas of your life. *Please answer all the*

*questions.* If you are unsure about which response to give to a question, please choose the one that appears most appropriate. This can often be your first response.

*Please keep in mind your standards, hopes, pleasures and concerns. We ask that you think about your life in the last two weeks. Please read each question, assess your feelings, and circle the number on the scale for each question that gives the best answer for you.*

|  | Very poor | Poor | Neither  poor nor  good | Good | Very good |
| --- | --- | --- | --- | --- | --- |
| **1** G1 How would you rate your quality of life? | 1 | 2 | 3 | 4 | 5 |

|  | Very  dissatisfied | Dissatisfied | Neither  satisfied nor  dissatisfied | Satisfied | Very  satisfied |
| --- | --- | --- | --- | --- | --- |
| **2** G4 How satisfied are you with your health? | 1 | 2 | 3 | 4 | 5 |

The following questions ask about **how much** you have experienced certain things in the last two weeks.

|  | Not at all | A little | A moderate  amount | Very much | Extremely |
| --- | --- | --- | --- | --- | --- |
| **3** To what extent do you feel that physical pain prevents you from doing what you need to do? | 1 | 2 | 3 | 4 | 5 |
| **4** How much do you need any medical treatment to function in your daily life? | 1 | 2 | 3 | 4 | 5 |
| **5** How much do you enjoy life? | 1 | 2 | 3 | 4 | 5 |
| **6** To what extent do you feel your life to be meaningful? | 1 | 2 | 3 | 4 | 5 |
| **7** How well are you able to concentrate? | 1 | 2 | 3 | 4 | 5 |
| **8** How safe do you feel in your daily life? | 1 | 2 | 3 | 4 | 5 |
| **9** How healthy is your physical environment? | 1 | 2 | 3 | 4 | 5 |

**The following questions ask about how completely you experience or were able to do certain things in the last two weeks.**

|  | Not at all | A little | Moderately | Mostly | Completely |
| --- | --- | --- | --- | --- | --- |
| **10** Do you have enough energy for everyday life? | 1 | 2 | 3 | 4 | 5 |
| **11** Are you able to accept your bodily appearance? | 1 | 2 | 3 | 4 | 5 |
| **12** Have you enough money to meet your needs? | 1 | 2 | 3 | 4 | 5 |
| **13** How available to you is the information that you need in your day-to-day life? | 1 | 2 | 3 | 4 | 5 |
| **14** To what extent do you have the opportunity for leisure activities? | 1 | 2 | 3 | 4 | 5 |

The following questions ask you to say how **good or satisfied** you have felt about various aspects of your life over the last two

weeks

|  | Very poor | Poor | Neither poor nor  good | Good | Very good |
| --- | --- | --- | --- | --- | --- |
| **15** How well are you able to get around? | 1 | 2 | 3 | 4 | 5 |

|  | Very  dissatisfied | Dissatisfied | Neither satisfied nor dissatisfied | Satisfied | Very  satisfied |
| --- | --- | --- | --- | --- | --- |
|  | 1 | 2 | 3 | 4 | 5 |
| **16** How satisfied are you with your sleep? | 1 | 2 | 3 | 4 | 5 |
| **17** How satisfied are you with your ability to perform your daily living activities? | 1 | 2 | 3 | 4 | 5 |
| **18** How satisfied are you with your capacity for work? | 1 | 2 | 3 | 4 | 5 |
| **19** How satisfied are you with yourself? | 1 | 2 | 3 | 4 | 5 |
| **20** How satisfied are you with your personal relationships other than your family? | 1 | 2 | 3 | 4 | 5 |
| **21** How satisfied are you with your sex life? | 1 | 2 | 3 | 4 | 5 |
| **22** How satisfied are you with the support you get from your friends? | 1 | 2 | 3 | 4 | 5 |
| **23** How satisfied are you with the conditions of your living place? | 1 | 2 | 3 | 4 | 5 |
| **24** How satisfied are you with your access to health services? | 1 | 2 | 3 | 4 | 5 |
| **25** How satisfied are you with your transport? | 1 | 2 | 3 | 4 | 5 |

The following question refers to **how often** you have felt or experienced certain things in the last two weeks.

|  | Never | Seldom | Quite often | Very often | Always |
| --- | --- | --- | --- | --- | --- |
| **26** How often do you have negative feelings such as blue mood, despair, anxiety, depression? | 1 | 2 | 3 | 4 | 5 |

|  | Not at all | A little | Moderately | Very much | Extremely |
| --- | --- | --- | --- | --- | --- |
| **U.27** To what extent are the difficulties in pressure and control about your relationships with people close to you in your life (wife, collaegue, relative)? | 1 | 2 | 3 | 4 | 5 |

Thank you for your help...
